# Contrasting assembly mechanisms and drivers of soil rare and abundant bacterial communities in 22-year continuous and non-continuous cropping systems

**DOI:** 10.1038/s41598-022-07285-2

**Published:** 2022-02-28

**Authors:** Yan Su, Yanxia Hu, Haiyun Zi, Yi Chen, Xiaopeng Deng, Binbin Hu, Yonglei Jiang

**Affiliations:** 1 Yunnan Academy of Tobacco Agricultural Sciences, Kunming, 650021 China; 2 Key Laboratory of Mountain Surface Processes and Ecological Regulation, Institute of Mountain Hazards and Environment, Chinese Academy of Sciences, Chengdu, 610041 China; 3 College of Biology and the Environment, Nanjing Forestry University, Nanjing, 210037 China; 4 University of Chinese Academy of Sciences, Beijing, 100039 China

**Keywords:** Bacteria, Biotechnology

## Abstract

Despite many studies on the influence of cropping practices on soil microbial community structure, little is known about ecological patterns of rare and abundant microbial communities in response to different tobacco cropping systems. Here, using the high-throughput sequencing technique, we investigated the impacts of two different cropping systems on soil biochemical properties and the microbial community composition of abundant and rare taxa and its driving factors in continuous and rotational tobacco cropping systems in the mountain lands of Yunnan, China. Our results showed that distinct co-occurrence patterns and driving forces for abundant and rare taxa across the different cropping systems. The abundant taxa were mainly constrained by stochastic processes in both cropping systems. In contrast, rare taxa in continuous cropping fields were mainly influenced by environmental perturbation (cropping practice), while governed by deterministic processes under rotational cropping. The α-diversity indices of rare taxa tended to be higher than those of the abundant ones in the two cropping systems. Furthermore, the network topologies of rare taxa were more complex than those of the abundant taxa in the two cropping systems. These results highlight that rare taxa rather than abundant ones play important roles in maintaining ecosystem diversity and sustaining the stability of ecosystem functions, especially in continuous cropping systems.

## Introduction

In China, the farmland area has been decreased approximately 300 × 10^4^ ha from 1996 to 2009 with the rapid development of industrialization and urbanization^1^. Tobacco (*Nicotiana tobacum* L.) is the major income source for local farmers in Yunnan^2^. However, the pattern of tobacco planting is mainly continuous cropping due to limited arable land, which leads to a decline in crops productivity, soil quality, and changes in soil microbe communities^3,4^. Gao et al.^5^ found that continuous cropping of Sweet Potato decreased the abundances of many beneficial microbes (e.g., *Arthrobacter*, *Lysobacter*, and *Chaetomium*), while increased the detrimental microbes (e.g., *Verticillium*, *Fusarium*, and *Colletotrichum*) in soils. In contrast to continuous cropping, crop rotation has been considered as an effective method to alleviate the harmful effects of continuous cropping by increasing the abundance and diversity of soil microbial communities^4^.

Traditionally, the majority of soil microbial studies focused on the abundant taxa^6^, whereas the effects of different farming systems on rare taxa were less explored. In fact, it is increasingly recognized that microbial communities are composed of a small number of abundant species with lower diversity but a great number of rare taxa with high diversity^7,8^. An increasing number of studies come to recognize that rare taxa contribute to the majority of genetic and functional diversity and play important roles in maintaining ecosystem functions^9–12^. Recent studies have shown that rare taxa may counteract environmental disturbances by increasing the functional redundancy of soil microbiota under environmental stress^13,14^, which remains to be empirically confirmed in different farming systems.

Rare taxa have a different response to environmental changes from abundant taxa^14,15^. Studies have observed that there were divergent assembly processes for abundant and rare taxa in oil-contaminated soils^15^ and in agricultural soils^16^. Deterministic processes refer to biotic and abiotic factors determine microbial communities, while stochastic processes regard that microbial communities are mainly governed by random changes^17,18^. Many studies show that environmental factors alter the relative influences of stochastic and deterministic assembly processes^19–22^. For example, abundant taxa have been found to be constrained by stochastic processes in the surface layer of the northwestern Pacific Ocean^23^, while rare taxa have been found to be governed by stochastic in oil-contaminated soils^15^. At present, it is still unclear the primary forces that influence the assembly of rare and abundant taxa in continuous tobacco cropping soils.

The aims of this study were (1) to determine whether abundant and rare taxa show different responses to crop rotation and continuous cropping practices, (2) to investigate which taxa play a major role in the resilience to the continuous cropping, i.e., abundant or rare taxa, (3) to explore the relative importance of environmental variables influencing the assembly of rare and abundant sub-communities in the crop rotation and continuous cropping systems. For this, based on high-throughput Illumina paired-end sequencing technologies we analyzed the bacterial small-subunit ribosomal RNA (16S rRNA) gene sequences to determine both rare and abundant bacterial lineages in continuous and rotational tobacco cropping fields in the Yunnan Province, China. We hypothesized that: (1) the abundant and rare taxa exhibit distinct responses and assemblage patterns in continuous and non-continuous tobacco soils; (2) the co-occurrence patterns of abundant and rare taxa would show divergence in both continuous and non-continuous tobacco soils, and their interactions will be weakened at the continuous tobacco fields due to continuous-cropping obstacle; (3) the underlying factors and driving forces regulating community assemblages vary across the continuous and non-continuous tobacco fields.

## Results

### Physiochemical properties in soils

Compared to continuous cropping, fine silt, coarse silt, pH, TOC, TN, TP, TK, NO_3_^−^-N, NH_4_^+^-N, DON, AP, AK, SOM, MBC, MBN, and MBP were significantly higher in crop rotation soils (p < 0.05), but the sand was markedly decreased (p < 0.05). However, there were no significant differences in MBC:N, MBC:P, and MBN:P between continuous cropping and crop rotation soils (Table 1).

**Table 1:** The soil physical–chemical and biological properties.

Treatments	Fine silt	Coarse silt	Sand	Soil pH	TOC	TN	TP	TK	NO_3_^–^-N	NH_4_^+^-N
	(%)	(%)	(%)		(g kg^−1^)	(g kg^−1^)	(g kg^−1^)	(g kg^−1^)	(mg kg^−1^)	(mg kg^−1^)
M	8.92 ± 0.45b	32.78 ± 0.17b	58.30 ± 0.39a	6.27 ± 0.23a	5.85 ± 0.26b	0.58 ± 0.07b	0.90 ± 0.04a	5.98 ± 0.20b	17.17 ± 3.36b	1.23 ± 0.07b
R	15.42 ± 0.43a	34.99 ± 0.14a	49.59 ± 0.50b	6.86 ± 0.09b	7.9 ± 0.27a	1.13 ± 0.12a	1.12 ± 0.1a	7.61 ± 0.37a	54.76 ± 11.08a	2.29 ± 0.34a

Treatments	DON	AP	AK	SOM	MBC	MBN	MBP	MBC:N	MBC:P	MBN:P
	(mg kg^−1^)	(mg kg^−1^)	(mg kg^−1^)	(g kg^−1^)	(mg kg^−1^)	(mg kg^-1^)	(mg kg^−1^)			
M	48.34 ± 3.66b	36.50 ± 0.84a	93.32 ± 14.56b	10.07 ± 0.45b	66.17 ± 4.36b	16.55 ± 1.28a	0.98 ± 0.04b	4.03 ± 0.32a	67.97 ± 5.21a	17.11 ± 2.11b
R	100.13 ± 12.18a	41.28 ± 3.23a	152.62 ± 48.17a	13.63 ± 0.46a	82.84 ± 1.85a	19.62 ± 1.67a	1.37 ± 0.01a	4.29 ± 0.37a	61.05 ± 3.37a	14.63 ± 2.23b

Mean ± standard deviation. Values within the same column followed by the same subscripted lower-case letters do not differ at *p* < 0.05. *TOC* total organic carbon, *TN* total nitrogen, *TP* total phosphorus, *TK* total potassium, *NO*_*3*_^*–*^*-N* nitrate nitrogen, *NH*_*4*_^*+*^*-N* ammonium nitrogen, *DON* dissolved organic nitrogen, *AP* available phosphorus, *AK* available potassium, *SOM* soil organic matter, *MBC* microbial biomass carbon, *MBN* microbial biomass nitrogen, *MBP* microbial biomass phosphorus, *M* tobacco monoculture, *R* tobacco-rice rotation.

### General distribution patterns of rare and abundant taxa

**Figure 1:**
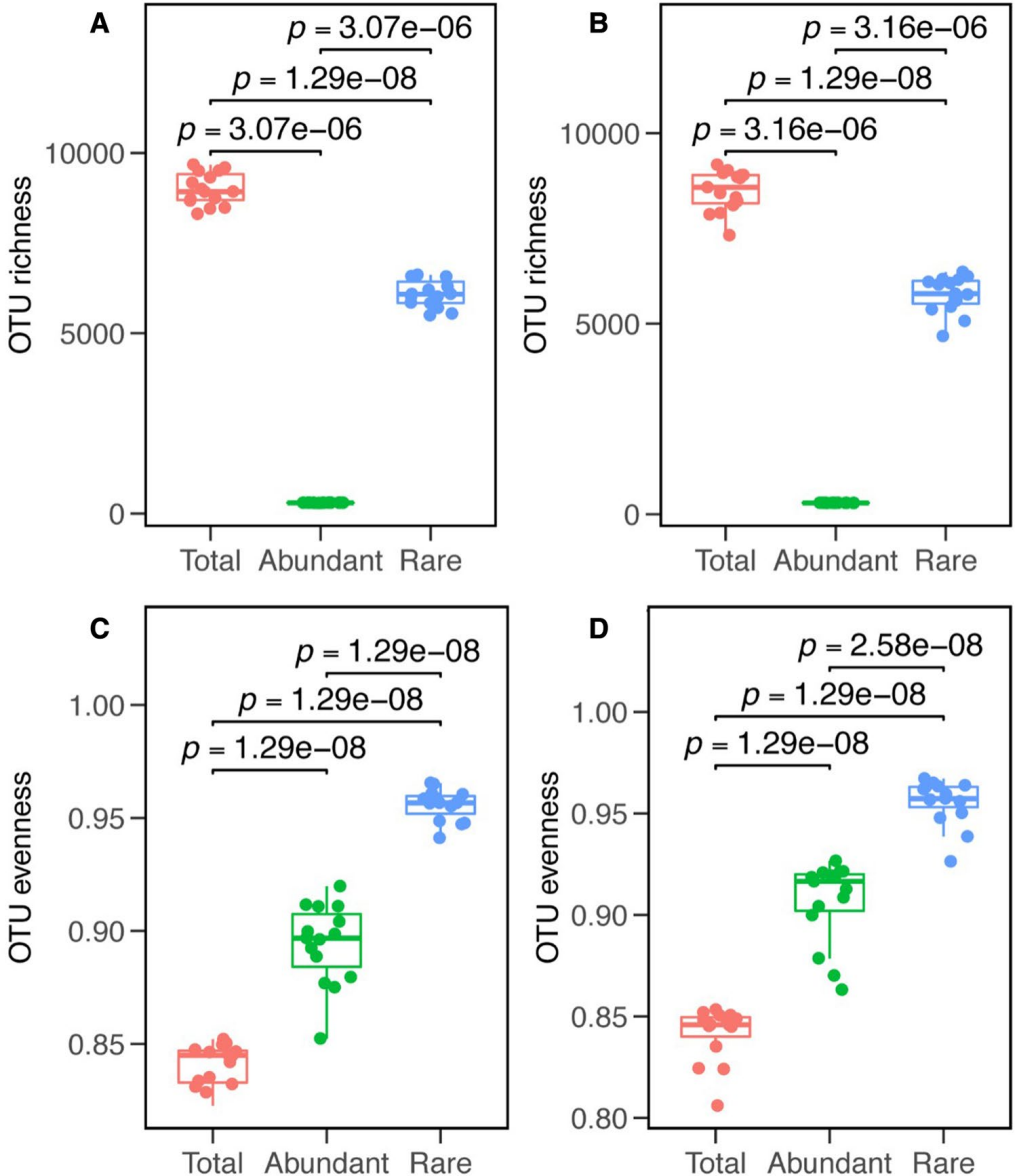
OTU richness (**A**) and Pielou’s evenness (**C**) of the total, abundant, and rare bacterial communities in the soil of tobacco monoculture and variation of OTU richness (**B**) and Pielou’s evenness (**D**) in the soil of tobacco-rice rotation. Box is drawn to represent values from lower 1/4 quartile to upper 1/4 quartile; *P* < 0.05 represent significant differences among the three taxa according to one-way ANOVA. *Total* whole bacterial communities; *Abundant* abundant taxa, *Rare* rare taxa.

**Figure 2:**
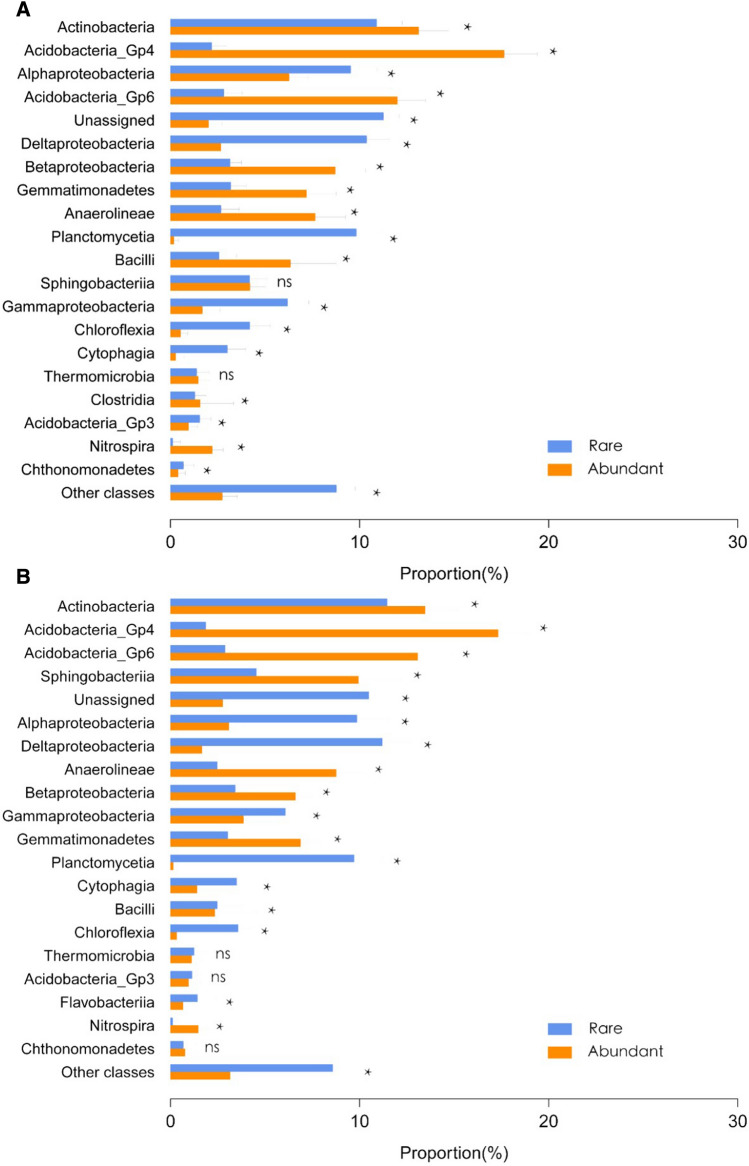
Taxonomic distributions of abundant and rare bacterial sub-communities at the class level in the soil of tobacco monoculture soil (**A**) and tobacco-rice rotation (**B**). *Abundant* abundant taxa, *Rare* rare taxa, *ns* not significant, *P < 0.05 (Wilcoxon Rank Sum tests); Rank the species from top to bottom according to their total relative abundance.

**Figure 3:**
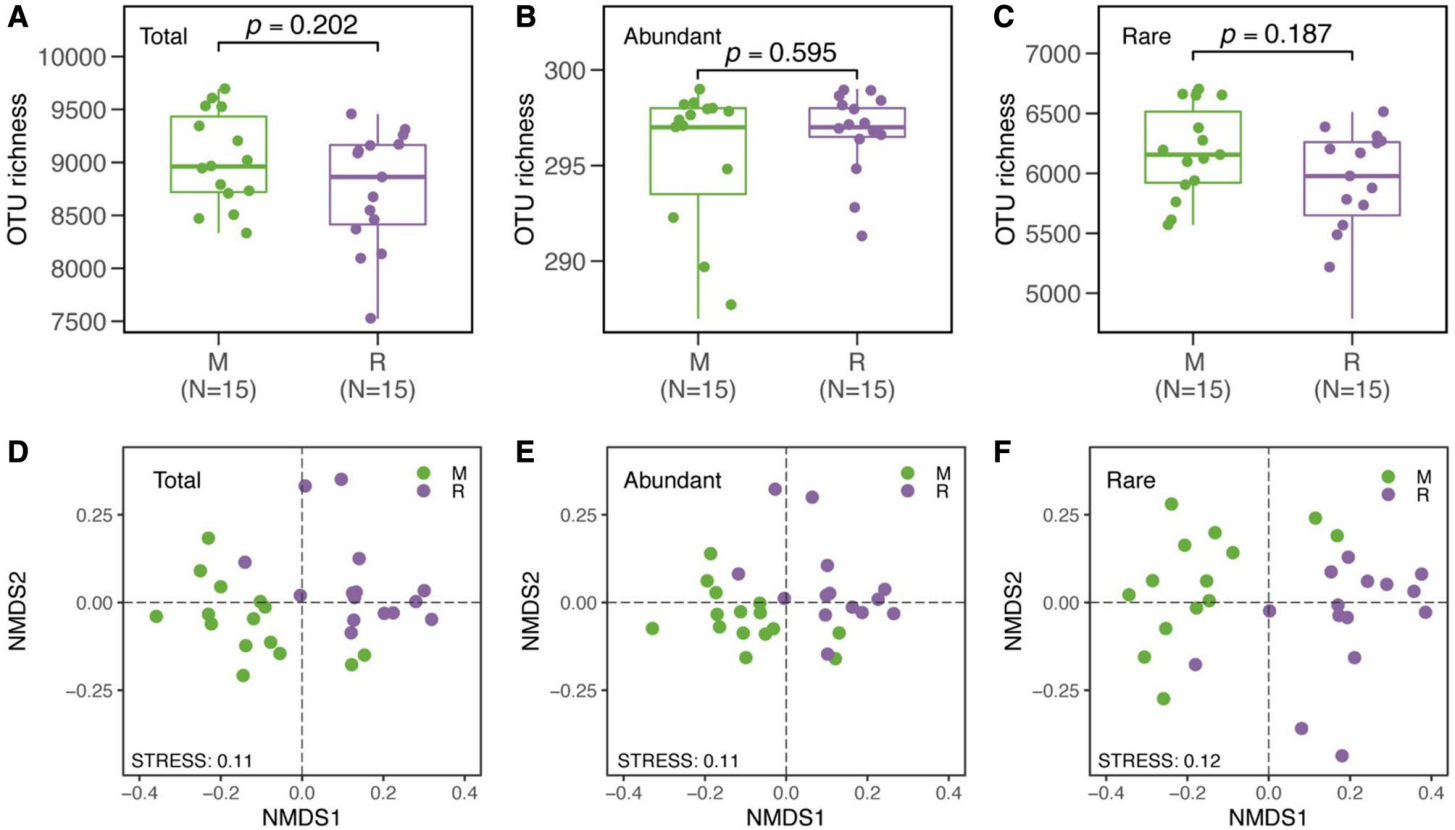
OTU richness of the total, abundant, and rare taxa in tobacco monoculture and tobacco-rice rotation (**A**–**C**); and bacterial community composition of the total, abundant, and rare taxa in tobacco monoculture and tobacco-rice rotation as indicated by non-metric multidimensional scaling (NMDS) (**D**–**F**). *Total* whole bacterial communities, *Abundant* abundant taxa, *Rare* rare taxa, *M* tobacco monoculture, *R* tobacco-rice rotation.

**Figure 4:**
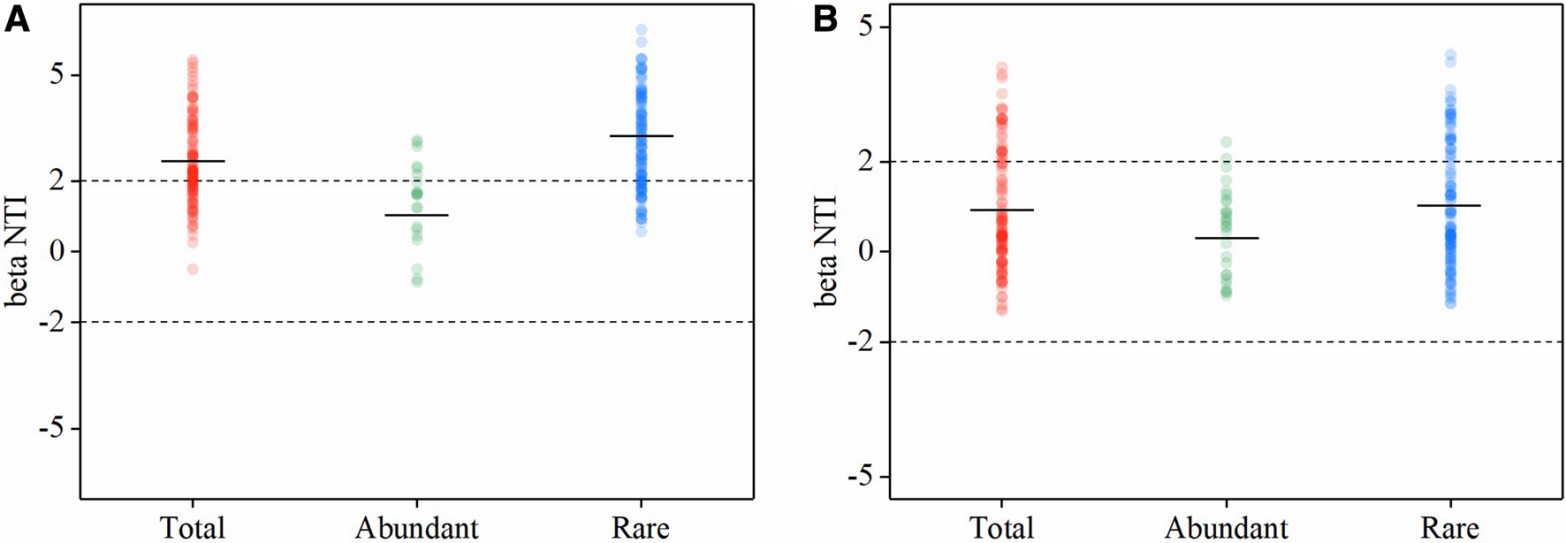
Distribution of beta Nearest Taxon Index (beta NTI) indicating assemblage processes of the total, abundant, and rare taxa in soil bacterial of tobacco monoculture (**A**) and tobacco-rice rotation (**B**). Black lines represent the median value of each taxa. Dash black lines (statistically significant if > 2 or < − 2) represent a 95% confidence interval around the expected neutral assembly. *Total* whole bacterial communities, *Abundant* abundant taxa, *Rare* rare taxa.

After quality filtering and the removal of chimeric sequences, the Illumina V4-V5 16S rRNA data set contained 1,460,302 quality sequences (range 124,400–477,309 sequence reads per sample) and 17,165 OTUs (Table S1). In both continuous cropping and crop rotation soils, the abundant taxa constituted a very low proportion of OTUs (mean 3.28% and 3.40%), but accounted for 41.30% and 41.81% of the average relative abundance in each sample. The rare taxa constituted a high proportion of the OTUs (mean 68.44% and 67.39% OTUs), while they contributed to an average of only 19.25% and 18.02% of the relative abundance in each sample (Table S1). The Goods coverage was 0.86 to 0.99, indicating that the number of sequence reads was sufficient to capture most taxa in each sample (Table 1). α-diversity (both the richness (Chao 1) and evenness (Shannon index) of the bacterial community in crop rotation soils tended to be higher than those in continuous cropping soils. The rare taxa showed higher α-diversity, both in terms of Chao1, Observed richness, and Shannon than the abundant taxa in the two cropping systems (Table 2). The OUT richness of rare taxa was significantly higher than that of the abundant taxa in both continuous cropping and crop rotation soils (Fig. 1A,B). The evenness of rare taxa and abundant taxa was significantly higher than that of the total taxa in both continuous cropping and crop rotation soils, and the evenness of rare taxa was the highest (Fig. 1C,D). In the continuous cropping, the top 5 most dominant phyla were Actinobacteria, Acidobacteria_Gp4, Alphaproteobacteria, Acidobacteria_Gp6, and Unassigned. The top 5 most dominant phyla in the crop rotation were Actinobacteria, Acidobacteria_Gp4, Acidobacteria_Gp6, Sphingobacteriia, and Unassigned. In both continuous cropping and crop rotation, the abundant taxa were more frequently Actinobacteria, Acidobacteria_Gp4, Acidobacteria_Gp6, Betaproteobacteria, Gemmatimonadetes, and Anaerolineae. The rare taxa were Alphaproteobacteria, Unassigned, Deltaproteobacteria, Planctomycetia, Gammaproteobacteria, and Chloroflexia. Acidobacteria_Gp3 and Chthonomonadetes were dominant in the rare sub-community in continuous cropping soils, but there was no significant difference in them between the rare and the abundant taxa in the rotation cropping soils. Bacilli dominated in the abundant taxa in continuous cropping soils, but it dominated in the rare taxa in crop rotation soils. There was no significant difference in Sphingobacteriia in the abundant and rare taxa in continuous cropping soils, but it dominated in the abundant and rare taxa in the crop rotation soils (Fig. 2). Different cropping modes had a significant influence on the abundance of total, abundant and rare taxa (Fig. 3A–C). The results of NMDS showed that the similarity of the total, abundant and rare taxa between continuous cropping and crop rotation was low (Fig. 3D–F). Rare taxa had higher community dissimilarity than abundant taxa in both cropping modes (Fig. 3F). Null model analysis showed that the influences of determinism (i.e. βNTI ≥ 2 or βNTI ≤ − 2) and stochasticity (i.e. − 2 < βNTI < 2) on assembly varied between the continuous cropping and crop rotation systems. Most of the β-NTI values of total and the rare taxa in the continuous cropping and crop rotation were higher than 2 (Fig. 4), while the β-NTI values of the abundant taxa were between− 2 and 2, indicating that the deterministic processes dominate the communities of the total and rare taxa in continuous cropping soils, while the random processes dominant in abundant taxa. In addition, the β-NTI values of the three sub-communities were between − 2 and 2 in crop rotation soils, indicating the three sub-communities were governed by the stochastic assembly in rotation soils. The β-NTI values of the total and rare taxa were higher than those of the abundant taxa (Fig. 4).

**Table 2:** The soil microbial diversity and abundance in different cropping patterns of tobacco.

Soil type	Observed richness	Chao1	Shannondiversity (H)	Simpson	Good’s coverage
**M**					
Whole	8747.7333 ± 550.98a	10,949.7639 ± 630.16a	7.6402 ± 0.16ab	0.0020 ± 0.0008ab	0.97 ± 0.0018a
Abundant	296.7333 ± 2.25c	297.3578 ± 2.41c	5.1616 ± 0.12b	0.0101 ± 0.003a	0.99 ± 0.00003a
Rare	5901.6667 ± 480.57b	8060.411 ± 560.18b	8.2959 ± 0.16a	0.0004 ± 0.0002b	0.86 ± 0.0157a
**R**					
Whole	9024.2667 ± 442.52a	11,339.1012 ± 522.74a	7.6541 ± 0.11ab	0.0022 ± 0.0005ab	0.97 ± 0.0016a
Abundant	295.3333 ± 4.03c	296.4167 ± 3.42c	5.0863 ± 0.10b	0.0117 ± 0.0024a	0.99 ± 0.00004a
Rare	6177.8 ± 379.33b	8441.8641 ± 482.06b	8.3415 ± 0.11a	0.0004 ± 0.0001b	0.86 ± 0.0165a

Different letters within the same column indicate significant differences at (*p* < 0.05). *M* tobacco monoculture, *R* tobacco-rice rotation, *Whole* whole bacterial communities, *Abundant* abundant taxa, *Rare* rare taxa.

### Co-occurrence patterns of the three sub-communities

The topological characteristics of the co-occurrence network showed that the nodes, modularity, clustering coefficient, average path length, network diameter, and average degree were all higher in the crop rotation soils than in the continuous cropping, while continuous cropping had more edges (Edges) (Table 3). Compared with those in continuous cropping, the rare taxa in the crop rotation soils had more nodes and edges as well as higher modularity, average path length, and network diameter (Table 3). The number of nodes, edges, modularity, and cluster coefficient of rare taxa was all higher, while the average path length, network diameter, and average degree were all lower than those of abundant taxa in both continuous cropping and crop rotation soils (Table 3). In addition, in Empirical networks, compared with the abundant taxa network, the rare taxa network was broader and had higher number of modules, modularity, and average geodesic distance (GD), while the abundant taxa had higher average connectivity (avgK) and average clustering coefficient (avgCC). In Random networks, GD and modularity in rare taxa networks were higher than those in the abundant taxa (Table 4).

**Table 3:** The soil bacterial network properties in different cropping patterns of tobacco.

Index	M			R		
	Total	Abundant	Rare	Total	Abundant	Rare
Nodes	395	224	349	401	220	363
Edges	2462	1034	1316	1631	872	1380
Modularity	0.435	0.404	0.698	0.629	0.449	0.761
Cluster coefficient	0.503	0.508	0.641	0.517	0.613	0.634
Average path length	2.669	2.556	2.131	2.973	2.619	2.234
Network diameter	9	7	7	10	8	8
Average degree	8.135	7.927	7.600	12.466	9.232	7.542

*M* tobacco monoculture, *R* tobacco-rice rotation, *Total* whole bacterial communities, *Abundant* abundant taxa, *Rare* rare taxa.

**Table 4:** Topological properties of the empirical molecular ecological networks (MENs) among whole, abundant and rare, and their associated random MENs in different cropping patterns of tobacco soil.

Soil type	Microbial community	Empirical networks							Random networks		
		Modules	Network size	Link	avgK	GD	avgCC	Modularity	GD ± SD	avgCC ± SD	Modularity ± SD
M	Whole	103	596	916	3.07	6.02	0.14	0.78	4.64 ± 0.06	0.01 ± 0.003	0.61 ± 0.006
	Abundant	18	191	592	6.20	4.98	0.37	0.55	3.08 ± 0.04	0.08 ± 0.01	0.34 ± 0.006
	Rare	56	343	366	2.13	7.13	0.04	0.83	6.17 ± 0.22	0.01 ± 0.004	0.79 ± 0.008
R	Whole	51	429	954	4.45	4.37	0.17	0.62	3.76 ± 0.04	0.03 ± 0.01	0.45 ± 0.006
	Abundant	19	163	770	9.45	3.73	0.41	0.37	2.69 ± 0.04	0.19 ± 0.014	0.23 ± 0.007
	Rare	53	352	455	2.59	5.40	0.02	0.77	4.92 ± 0.12	0.01 ± 0.004	0.68 ± 0.008

*Network size* the number of OTUs (e.g., nodes) in a network, *avgK* average connectivity, *GD* average geodesic distance, *avgCC* average clustering coefficient, *M* tobacco monoculture, *R* tobacco-rice rotation, *Whole* whole bacterial communities, *Abundant* abundant taxa, *Rare* rare taxa.

**Figure 5:**
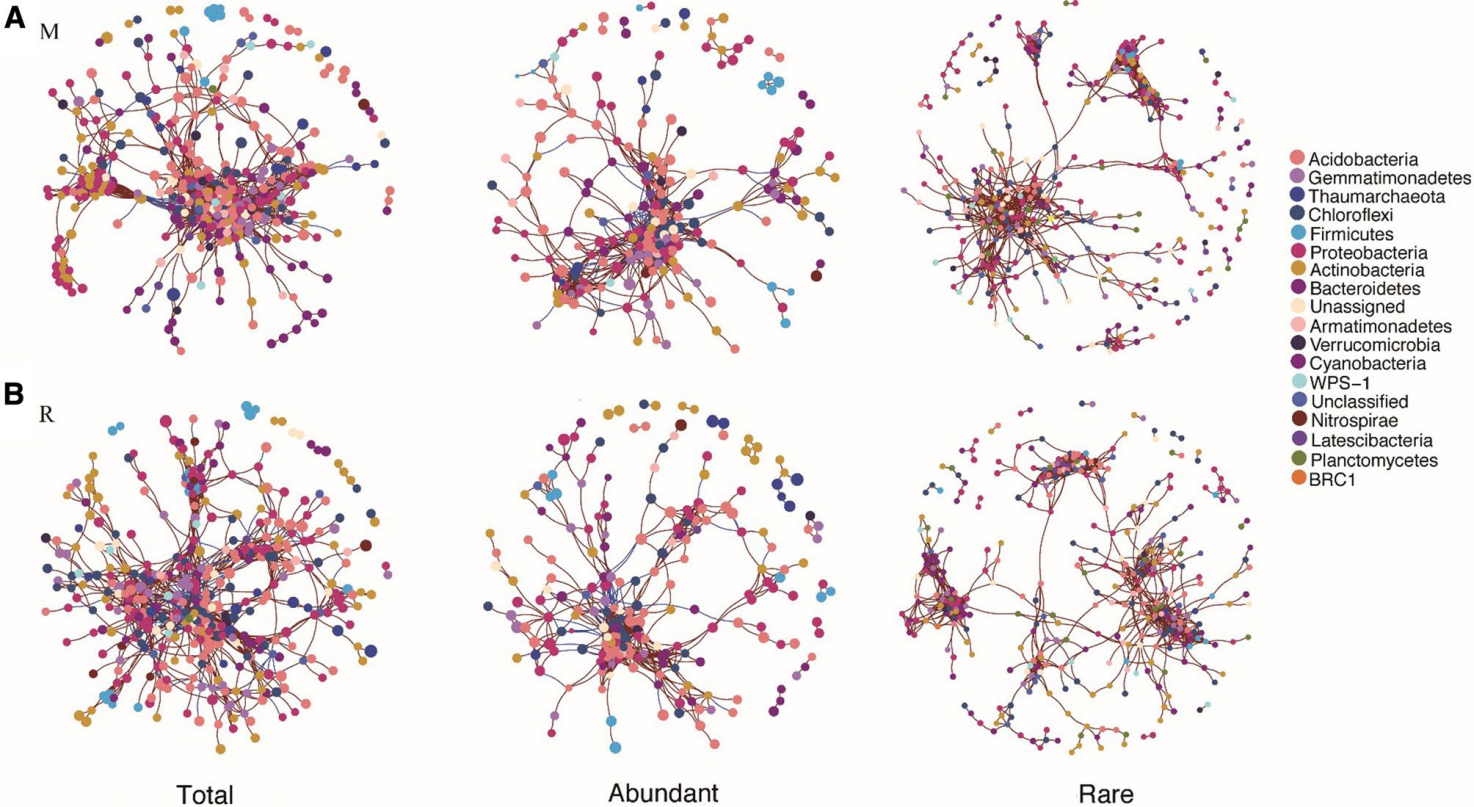
Co-occurrence network analysis of bacterial communities for the total, abundant and rare species (class level) in soil of tobacco monoculture (**A**) and tobacco-rice rotation (**B**). The red and blue colour of each connection between two nodes is positive and negative relationships of Spearman’s correlation coefficients.

In the two cropping modes, the nodes in the network graph mainly belong to 10 phyla: Acidobacteria, Gemmatimonadetes, Actinobacteria, Bacteroidetes, Chloroflexi, Plantctomycetes, and Proteobacteria (Fig. 5). The total taxa were more complex, while the abundant and rare taxa were more discrete in cropping rotation soils than those in continuous cropping soils. Compared with the abundant taxa, the rare taxa were more complex, as their structure was wider, and they had more nodes and edges and modularity in both continuous cropping and crop rotation soils (Fig. 5).

In the co-occurrence network of soil bacteria, most of the OTUs were divided into peripheral nodes (Peripherals) (Fig. S1). In continuous cropping soils, module hubs mainly belong to Acidobacteria_Gp4, Acidobacteria, Gemmatimonadales, Acidobacteria_Gp6, Deltaproteobacteria, Betaproteobacteria, Sphingobacteria, and Alphaproteobacteria, and most of these nodes had high intra-module connectivity (Zi) but the connectivity between modules (Pi) was low. In addition, connectors in the continuous cropping mainly belong to Actinobacteria, Acidobactena_Gp4, candidate_division, and Chloroflexi. These nodes had high inter-module connectivity (Pi) and low intra-module connectivity (Zi) (Fig. S1).

In crop rotation soils, OTUs classified as module hubs mainly belong to Alphaproteobactena, Acidobacteria_Gp6, Latescibacteria, Betaproteobacteria, Unassigned, candidate_division, Proteobacteria, Dellaproteobactenia, Proteobacteria and Acidobacteria. The OUTs belonging to Alphaproteobactena, Acidobacteria_Gp6, Betaproteobacteria, Proteobacteria, Dellaproteobactenia, and Proteobacteria not only had high intra-module connectivity (Zi) but also had high inter-module connectivity (Pi). The OUTs classified as connectors in the crop rotation soils were mainly Armatimonadetes, Armatimonadetes, Chloroflexi, Ignavibacteriae, Bacteroidetes, and Acidobacteria (Fig. S1).

### Relationship between microbial sub-communities and soil properties

**Figure 6:**
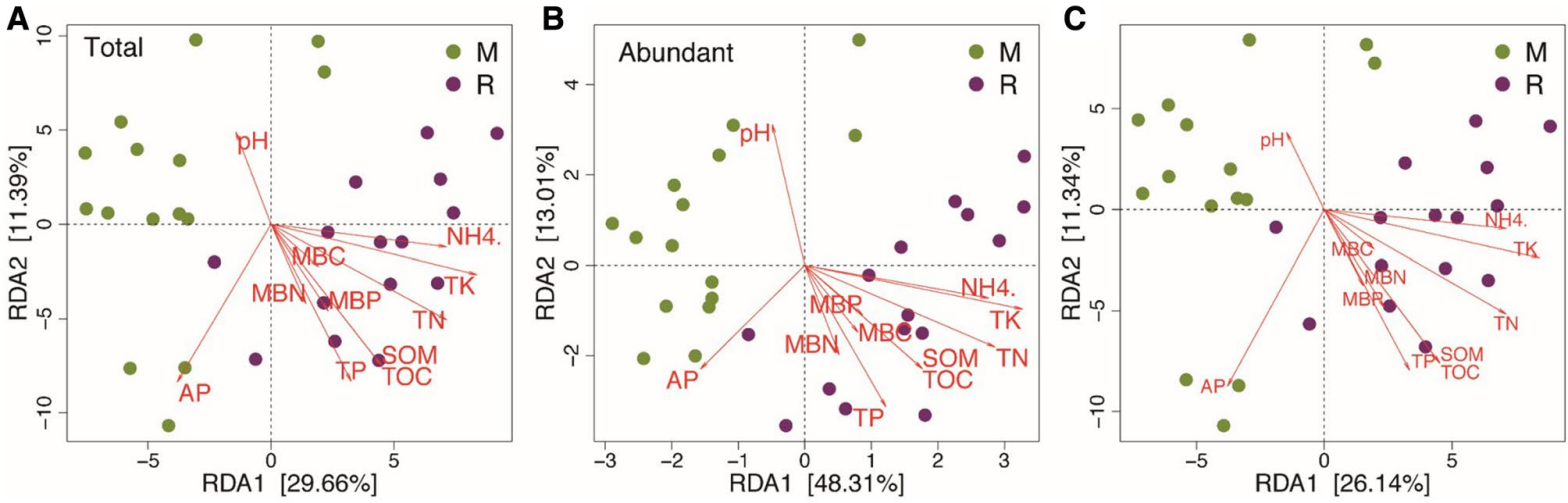
Redundancy analysis (RDA) ordination biplots of the total (**A**), abundant (**B**) and rare (**C**) bacterial community in relation to soil environmental variables. Arrows indicate directions of maximum variation of environmental variables; length of arrows indicates their importance. Each circle colored by two crop management represents the bacterial community for each sample. *TOC* total organic carbon, *TN* total nitrogen, *TP* total phosphorus, *TK* total potassium, *NO*_*3*_^*–*^*-N* nitrate nitrogen, *NH*_*4*_^*+*^*-N* ammonium nitrogen, *AP* available phosphorus, *SOM* soil organic matter, *MBC* microbial biomass carbon, *MBN* microbial biomass nitrogen, *MBP* microbial biomass phosphorus, *M* tobacco monoculture, *R* tobacco-rice rotation.

The first two axes of the RDA analysis accounted for 29.66% and 11.39% of the variance in the total taxa, 48.31% and 13.01% in the abundant taxa, and 26.14% and 11.34% in the rare taxa. Among all the soil properties involved in the RDA analysis, AP, TOC, SOM, TP, TN, and TK were closely related to the soil bacterial communities in the two cropping patterns (Fig. 6). The Mantel test results indicated that the total microbial taxa were correlated with soil pH, TOC, SOM, TN, TP, TK, NH_4_^+^-N, N0_3_^–^-N, AP, MBN, and MBP in both continuous cropping and crop rotation soils (Table 5). In continuous cropping system, abundant microbial taxa were connected with TOC, SOM, TN, MBN, and MBC, however rare taxa showed significant correlations with soil pH, TP, TK, NH_4_^+^-N, NO_3_^–^-N and AP. The rare taxa are mainly connected to soil pH, SOM, TP, AP, NH_4_^+^-N, N0_3_^–^-N, MBN, and MBP in crop rotation soils. Both rare and abundant microbial taxa showed marked correlations with TOC, TK, and MBN, but the former exhibited stronger correlations with them than the latter (Table 5).

**Table 5:** Spearman’s correlations of soil microbial community and physiochemical factors in different cropping patterns of tobacco, based on Mantel tests.

Soil properties	M			R		
	Whole	Abundant	Rare	Whole	Abundant	Rare
pH	0.602**	0.132	0.724**	0.756**	0.172	0.657**
TOC (g kg^−1^)	0.685**	0.342*	0.486*	0.753**	0.325*	0.529**
SOM (g kg^−1^)	0.602**	0.328*	0.039	0.681**	0.212	0.453*
TN (g kg^−1^)	0.742**	0.433*	0.487*	0.759**	0.364*	0.050
TP (g kg^−1^)	0.532*	0.206	0.428*	0.549*	0.014	0.457*
TK (g kg^−1^)	0.534*	0.301	0.349*	0.654**	0.365*	0.724**
NO_3_^–^-N (mg kg^−1^)	0.818**	0.258	0.619**	0.865**	0.019	0.784**
NH_4_^+^-N (mg kg^−1^)	0.827**	0.301	0.436*	0.804**	0.021	0.449*
AP (mg kg^−1^)	0.827**	0.301	0.436*	0.804**	0.021	0.449*
MBC (mg kg^−1^)	0.027	0.031	0.127	0.256	0.246	0.534
MBN (mg kg^−1^)	0.427*	0.606**	0.148	0.753**	0.456*	0.631**
MBP (mg kg^−1^)	0.629**	0.506*	0.206	0.699**	0.068	0.536*

*TOC* total organic carbon, *SOM* soil organic matter, *TN* total nitrogen, *TP* total phosphorus, *TK* total potassium, *NO*_*3*_^*–*^*-N* nitrate nitrogen, *NH*_*4*_^*+*^*-N* ammonium nitrogen, *AP* available phosphorus, *MBC* microbial biomass carbon, *MBN* microbial biomass nitrogen, *MBP* microbial biomass phosphorus, *M* tobacco monoculture, *R* tobacco-rice rotation, *Whole* whole bacterial communities, *Abundant* abundant taxa, *Rare* rare taxa. Bold text indicates statistical significance.

*P < 0.05 significance level.

**P < 0.01 significance level.

****P < 0.001 significance level.

## Discussion

Studies have revealed that different cropping systems can significantly change the physicochemical properties of soil. Continuous cropping leads to the deterioration of soil chemical properties and crop yield reduction^24,25^, while crop rotation could improve soil properties and soil microbial community^26,27^. We also found that the soil properties and microbial parameters were greater in crop rotation soil than the continuous cropping soil. As the soil TN, TP, TK, and all available nutrients (NH_4_^+^-N, NO_3_^–^-N, AP, and AK) were obviously higher in crop rotation systems, which may relate to the change of soil pH under different cropping systems. Acidification could reduce the availability of nutrients and thereby reduce soil organism activity and biomass in soil^28–30^. Moreover, the improvement of soil texture and increased soil microorganisms are also tightly related to the enrichment in soil nutrient availability^31,32^. This indicates that soil nutrient contents were improved under crop rotation systems.

We found that the bacterial diversity was also greater in crop rotation soil than continuous cropping soil, and such responses may be linked to the soil physicochemical properties (e.g. soil pH, moisture, and nutrients content)^33,34^. As environmental variation is a key decisive factor of the distribution and abundance of microbes^35^. The relatively lower soil pH may decrease the bacterial diversity under continuous cropping systems, as soil acidity could change the stability of bacterial cell membranes and thus inhibits bacterial growth^36,37^. In addition, the high content of soil organic C in crop rotation systems, to some extent, likely influences bacterial diversity, activities, and community compositions. A meta-analysis from Venter et al.^38^ also revealed that increased microbial richness and diversity with crop rotation. In our study, soil microbial communities were further classified into rare and abundant microbial taxa. In general, abundant taxa had a higher capacity in competing for nutrient resources and thus not sensitive to environmental changes, and the opposite is true for rare taxa^39,40^. However, our results showed that the rare sub-communities have higher richness and α-diversity than the abundant taxa in both continuous cropping and crop rotation systems, indicating that rare taxa were, to some extent, not sensitive to the different cropping patterns, compared with abundant taxa^13,41^. The reason may be that the rare taxa may contribute to the resilience of the microbial community and sustain community function as an insurance source in continuous cropping soils due to their high functional redundancy and resistance to stress^14,40^. Especially, continuous cropping has strong negative effects on abundant taxa. Furthermore, greater community dissimilarity across the cropping systems was detected in the rare biosphere than in the abundant and total taxa, indicating the rare taxa may have a flexible taxonomic profile^11^.

Actinobacteria, Acidobacteria_Gp4, Alphaproteobacteria, Acidobacteria_Gp6 were the predominant bacterial phyla in all the soil samples, which are known to be among the dominating phyla in different agricultural practices^42^. Previous studies suggest that Actinobacteria is beneficial bacteria for plant growth^43^. Some research showed that the abundance of Actinobacteria decreased in continuous cropping soils^44^ but increased in a crop rotation system^45^. Proteobacteria and Actinobacteria are copiotrophic in nature, which is known to have high nutritional requirements^46,47^, whereas Acidobacteria belongs to oligotroph and prefers low nutrient availability and low pH^47,48^. Accordingly, we expect that the relative abundance of Acidobacteria increases while Actinobacteria declines in continuous cropping soils. This was not the case in our study. The relative abundance of Sphingobacterii was observed only in crop rotation soils, indicating that it was sensitive to soil environmental disturbances such as N and pH. In terms of both number of OTUs and sequences, the dominant phyla between the abundant and rare sub-community were obviously different (Fig. 2), and more unclassified bacteria were found in the rare taxa. This result was consistent with previous studies in other ecosystems^49,50^. However, Ho et al.^47^ found that Copiotrophic Actinobacteria was presented at higher relative abundances in abundant than in rare sub-communities. Nevertheless, this was not the case in our study. This inconsistency may be due to the difference in environmental gradients^31,51^ and geography^8,52^.

Nowadays, the β-Nearest Taxon Index (βNTI) was broadly used to predict the relative effects of deterministic and stochastic processes on microbial assemblages in microbial ecology^53,54^. Null model analysis revealed that the three sub-communities were driven by stochastic processes in crop rotation soils, while the total and rare microbial taxa were primarily driven by deterministic processes (Fig. 4). Previous results from Jiao et al.^35^ and Zheng et al.^55^ found that rare microbial taxa were generally affected by deterministic processes in agricultural soils. However, Du et al.^56^ thought that rare taxa were more limited by stochastic processes in soil. These inconsistent results may be explained by the different soil environments investigated. We speculate the inconsistency results in our study under different cropping systems may result from the neutral soil pH under crop rotation soils are suitable for most soil microbes, and caused environmental filtering weakened, resulting in the structuring of microbial communities was more influenced by stochastic processes rather than deterministic processes. The variation in environmental factors such as soil N and pH can influence the balance between stochastic and deterministic assembly processes^19^. Wang et al.^57^ reported that low N limits the microbial dispersal, and stochastic processes may overwhelm deterministic processes under environmental variation. Future efforts exploring the mechanism of soil environmental factors in driving assemble of soil microbial communities may further proceed.

Compared with the simple description of community structure and diversity indices, network analyses can provide in-depth information on microbial sub-community interactions^14^. In line with our hypothesis, the topological features of abundant and rare taxa in the crop rotation soils were larger than those in continuous cropping soils, except edge, and the results were in line with those of Liu et al.^58^ who reported that the co-occurrence network of crop rotation system was more complex than short-term cropping system. This may be due to the plant diversity and the plant residue increase in crop rotation systems, which have an influence on soil microbial communities by altering the exudates secreted by preceding crops^58,59^. This indicates that the crop rotation system has greater co-occurrence networks and more microbial taxa involved in the potential microbial interactions, and can better buffer the environmental changes^60,61^. Jiao et al.^15^ reported that abundant rather than rare taxa played vital roles in the construction of microbial networks in oil soils. However, Liu et al.^58^ reported that the microbial species that were defined as keystone taxa were not the richest OTUs in the soil under different cropping systems. Nevertheless, in our study, rare taxa in both continuous cropping and crop rotation systems had more connections and closer intra-associations, suggesting rare taxa may exert a great influence on microbial co-occurrences and constitute a more stable community than the abundant biosphere^62^. Overall, these findings indicate that rare species play a unique role in maintaining the function and stability of soil ecosystem.

Environmental factors made different contributions to the assembly of the total, abundant and rare microbial sub-communities in the different cropping soil. The redundancy analysis indicated that within the continuous cropping and crop rotation soils, the total, abundant and rare microbial taxa were well separated (Fig. 3). This finding suggests that cultivation pattern was a main diver in determining the shifts of microbial communities. Previous findings reported by Liu et al.^58^ also found that the community of soil microbes was different in response to continuous cropping and crop rotation systems, and the corresponding variations were partially connected with the changes of soil chemical properties. In our study, the variation of bacterial community composition was mainly driven by AP, TOC, SOM, TP, TN, TK, NH_4_^+^-N, and pH (Fig. 6). Similar conclusions have been drawn by Lauber et al.^63^. and Ai et al.^64^. Our study showed that soil properties made different contributions to the assembly of the rare and abundant microbial taxa in the two different cropping systems (Table 5). The rare microbial taxa were more connected with soil pH and soil nutrients than the abundant one (Table 5). Soil pH plays the dominant role in determining the soil microbial community structure with different cultivation patterns have been widely reported^51,58^. According to the results reported by Delgado-Baquerizo et al.^65^, the bacterial diversity and composition were partly driven by variation in soil nutrients at a regional scale. This suggests that long-term different agricultural cropping practices can act as a kind of environmental filtering, which can have a strongly impact on soil biotic communities in diverse manners.

## Conclusions

Our results showed that the rare and abundant taxa exhibited distinct responses to the different cropping systems. The rare taxa assembly was driven by deterministic processes in continuous cropping soils and by stochastic processes in crop rotation, while the abundant sub-community was driven primarily by stochastic processes in both cropping systems. The α-diversity indices of rare taxa tended to be higher than those of the abundant ones in the two cropping systems. Moreover, the network topologies of rare taxa were more complex than those of abundant taxa in the two cropping systems. Our results suggested that rare taxa functioned as the majority of microbial diversity to enhance bacterial resilience and resistance to continuous cropping disturbances and they served as an important role in maintaining the stability in the two different cropping systems. Our study highlights the ecological importance of rare taxa, which can be harnessed in the future for sustainable agriculture production.

## Materials and methods

### Study sites and sampling locations

This research was carried out in Yunnan Academy of Tobacco Agricultural Sciences' Yanhe Research Farm, Yunnan Province, China (24.14°N, 102.30°E). The study sites are characterized by red soil (classified as Ultisol in USDA (2014) soil taxonomy), which is the dominant type soil for flue-tobacco production in Yunnan^2^. The cultivar for flue-cured tobacco was K326. The mean annual precipitation in this area is about 1160 mm, most between June and October, and the mean annual temperature is 15.9 °C. The altitude in this area is 1680 m, with 2072 h of annual average sunshine.

The soil samplings were collected from two tobacco management systems: soil with tobacco monoculture and soil with tobacco-rice rotation. For the continuous cropping soils, the study site was established in 1998, and it has been continuous cropping tobacco for more than 22 years without rotation with other crops. For the non-continuous cropping soils, the fields were 1 year for tobacco planting and another year for rice cultivation. The two fields were annually added with appropriate chemical NPK fertilizers (97.5 kg ha^−1^, N: P_2_O_5_: K_2_O = 1:1:2.5). Other agronomic management measures were same for continuous and non-continuous cropping tobacco fields.

In August 2019, the soil samples were collected from 30 plots (3 × 10 m rectangular plot) after harvesting flue-cured tobacco. Five soil cores from the tillage layer (0–15 cm depth) were collected from each plot using a 5-cm diameter soil corer after removing surface material by hand, and mixed thoroughly and passed through a 2-mm sieve as a single sample. Therefore, there are fifteen soil samples for continuous and non-continuous cropping tobacco fields, respectively. The obtained soil samples were separated into two subsamples: (1) one portion for soil microbial DNA extraction and soil microbial biomass determinations (stored at − 80 °C), (2) the other portion for soil physical and chemical properties determinations. Soil texture, pH, soil organic matter (SOM), total organic carbon (TOC), total nitrogen (TN), total phosphorus (TP), total potassium (TK), dissolved organic nitrogen (DON), nitrate-nitrogen (NO_3_^–^-N), ammonium-nitrogen (NH_4_^+^-N), available phosphorus (AP) and available potassium (AK), microbial biomass carbon (MBC), microbial biomass nitrogen (MBN), and microbial biomass phosphorus (MBP) were tested using standard methods^66,67^.

### DNA extraction, 16S rRNA gene sequencing and sequence processing

Briefly, the total soil genomic DNA was extracted from 0.25 g of soil using the PowerSoil® DNA Isolation Kit (MoBio Laboratories, Inc., Carlsbad, USA) according to the manufacturer’s protocol. The extracted DNA was stored at − 80 °C until used for Illumina MiSeq sequencing. The bacteria-specific 16S rRNA primers 515F (5′-GTGCCAGCMGCCGCGG-3′) and 907R (5′-CCGTCAATTCMTTTRAGTTT-3′) were used to amplify the V4–V5 hypervariable regions of bacterial 16S rRNA genes^68^. The amplified PCR products were sequenced on Illumina MiSeq PE300 platform (Illumina Inc., San Diego, CA, USA) at Genesky Biotechnologies Inc., Shanghai, China using 250-bp paired-end reads. The sequencing analysis was as described previously^17,69^. Detailed methods of DNA extraction and purification were available in Jiang et al.^69^.

Raw Illumina paired-end reads were assembled with FLASH 1.2.7^70^, and sequence processing and quality filtering of reads were performed using the pyrosequencing pipeline tools from the QIIME (http://qiime.sourceforge.net/)^71^. Reads less than 200 bp and phred score < 25 were discarded, and the high-quality sequences were picked out by the USEARCH using UPARSE software package (http://drive5.com/uparse/)^72^. The high-quality sequences with 3% dissimilarity were split into the same operational taxonomic units (OTUs) by the UPARSE pipeline^72^. Subsequently, the representative sequence from each OTU was aligned by PyNAST, and each OTU taxonomic identity was predicted by the ribosomal database project (RDP) classifier using the Greengenes database. Furthermore, the OTUs that contained < 2 reads and singletons were eliminated and all samples were rarefied to 1,244,000 sequences per sample for further analysis. The raw sequence data reported in this paper have been deposited in the Genome Sequence Archive (Genomics, Proteomics & Bioinformatics 2017) in National Genomics Data Center (Nucleic Acids Res 2020), Beijing Institute of Genomics (China National Center for Bioinformation), Chinese Academy of Sciences, under accession number CRA003510 that are publicly accessible at https://bigd.big.ac.cn/gsa.

### Definition of abundant and rare taxa

The abundant and rare OTUs were defined following our previous publications that referred to their local (one sample) and regional (across samples) relative abundances^68^. At the local level (i.e., in one sample), locally abundant operational taxonomic units (OTUs) were defined as the OTUs with relative abundances > 0.1% of the total sequences within a sample, whereas locally rare OTUs were defined as their abundances were < 0.1% within a sample^39^. At the regional level (i.e., across all samples), regionally abundant and rare taxa were defined as OTUs with average relative abundances above and below 0.01%, respectively^17,73^. Finally, the downstream analyses were performed at three levels: whole OTUs, abundant, and rare OTUs.

### Sequencing data analysis

Both bacterial α-diversity indices [e.g., the OTU richness and Shannon diversity (*H*)] and β-diversity based on Bray–Curtis distance between samples were calculated by QIIME to estimate the ecological relationships of bacterial taxa within and among samples, respectively. Furthermore, the same number of sequences from each sample (1,244,000 reads per sample for bacteria) to avoid the effects of different sequencing depths. Nonmetric multidimensional scaling (NMDS) with Bray–Curtis dissimilarities was used to show the differences in the whole, abundant and rare bacterial communities between the continuous and non-continuous cropping tobacco soils using the R vegan package. The microbial community dissimilarities between the continuous and non-continuous cropping tobacco soils were estimated. The distributions of rare and abundant bacteria were calculated by Wilcoxon rank-sum tests. Furthermore, the *P*-values were corrected using the false discovery rate (FDR) correction in all statistical analyses. The microbial phylogenetic assembly on a within-community scale was estimated by the nearest-taxon index (NTI) according to the null model in the *Picante* package^74,75^. The high or positive NTI values indicate taxa clustering across the overall phylogeny, while low or negative NTI values represent overdispersion of taxa across the phylogeny^76^. On the other hand, the β-NTI index was used to estimate the microbial assembly processes^8^. According to Webb et al.^77^, the β-NTI measures the deviation of the β-mean nearest taxon distance (β-MNTD) from the β-MNTD of the null model, and this was calculated in Phylocom v42. Furthermore, values of |β-NTI| > 2 and |β-NTI| < 2 indicate a community that is dominated by deterministic processes and stochastic processes, respectively^8,53^.

The bacterial co-occurrence patterns of the continuous and non-continuous cropping soils were assessed by network analysis, and networks were visualized according to the interactive Gephi platform (https://gephi.org/)^22,39^. At the regional level (i.e., across all samples), the co-occurrence networks of rare and abundant bacterial taxa were built based on the correlation analysis for the continuous and rotational cropping soils as described by Jiao et al.^39^. Firstly, we calculated all pair-wise Spearman’s correlations between any two OTUs before constructing networks. Secondly, we cut off the two OTUs with Spearman’s correlation coefficients less than 0.6 and FDR-corrected *p*-values more than 0.01 to construct the networks. Each node represents one OUT, and each edge in the network represents significant correlations between two nodes. The node-level topology features were assessed by degree, betweenness, closeness, and eigenvector centrality. The network-level topology features were estimated by a set of indexes: the average path length, network diameter, average degree, graph density, clustering coefficient, and modularity^22^.

The phylogenetic molecular ecological networks of species (pMENs) for the continuous and non-continuous cropping soils were estimated and analyzed by the Molecular Ecological Network Analysis Pipeline (http://ieg2.ou.edu/MENA)^78^ Detailed information about the network theories, including algorithms, pipeline structure, and operational procedures were described in detail by Deng et al.^78^ and Zhou et al.^79^. The major pMENs network properties are as follows: the average geodesic distance (GD), average connectivity (avgK), and average clustering coefficient (avgCC), which were used to test differences among bacterial sub-communities. Besides, the network stability was estimated by the modularity index. The topological roles of nodes were characterized by their positions (within-module connectivity (Zi) and among-module connectivity (Pi))^68^.

Redundancy discriminatory analysis (RDA) was performed to calculate the influence of the explanatory variables on the bacterial communities following the forward selection procedure^80^. The correlations between bacterial communities (based on Bray–Curtis community dissimilarity) and soil physicochemical characteristics were assessed by Mantel tests.

All data analyses were performed with R (version 3.3.3; http://www.r-project.org) in R Studio (version 1.0.44; http://rstudio.org).

### Approval for plant experiments

Experimental research and field studies on plants (either cultivated or wild), including the collection of plant material, must comply with relevant institutional, national, and international guidelines and legislation.

## Supplementary Information

Supplementary Information.
