# Supplementary material for: Contrasting assembly mechanisms and drivers of soil rare and abundant bacterial communities in 22-year continuous and non-continuous cropping systems
Source: Sci Rep. 2022 Feb 28;12:3264. doi: 10.1038/s41598-022-07285-2 (PMC8885686; doi:10.1038/s41598-022-07285-2)
Supplement: Supplementary file 1 — Supplementary Information. [file 41598_2022_7285_MOESM1_ESM.docx]

**Contrasting assembly mechanisms and drivers of soil rare and abundant bacterial communities in 22-year continuous and non-continuous cropping systems**

Yan Su^1,2,4#^, Yanxia Hu^1#^, Haiyun Zi^3^, Yi Chen^1^, Xiaopeng Deng^1^, Binbin Hu^1^, and Yonglei Jiang^1,*^

^1^Yunnan Academy of Tobacco Agricultural Sciences, Kunming, China 650021

^2^Key Laboratory of Mountain Surface Processes and Ecological Regulation, Institute of Mountain Hazards and Environment, Chinese Academy of Sciences, Chengdu 610041, China

^3^College of Biology and the Environment, Nanjing Forestry University, Nanjing 210037, China

^4^University of Chinese Academy of Sciences, Beijing 100039, China

* Correspondence address:

Yonglei Jiang

Yunnan Academy of Tobacco Agricultural Sciences, Kunming, China 650021

E-mail: jiangyatas@163.com; Tel: +86-18388136300; Fax: +86-28-85222258

^#^These authors have contributed equally to this work and share ﬁrst authorship

**Supplementary information**

**Supplementary figures legends**

Figure S1 Zi-Pi plot showing the keystone species in the bacterial network, (A) tobacco monoculture；（B）tobacco-rice rotation. Each symbol represents an OTU. The threshold values of Zi and Pi for categorizing OTUs were 2.5 and 0.62, respectively.





Figure S1

Table S1. General description of community sequencing and rare and abundant OTU datasets from soil bacterial in tobacco monoculture and tobacco-rice rotation.

| **Samples** | **Sites** | **OTUs** | **Rare OTUs (<0.005%)** | **Abundant OTUs (>0.05%)** | **%Rare OTUs** | **%Abundant OTUs** | **Abundance of rare OTUs (%)** | **Abundance of abundant OTUs (%)** |
| --- | --- | --- | --- | --- | --- | --- | --- | --- |
| 1 | M | 9610 | 6653 | 299 | 69.23 | 3.11 | 19.74 | 40.58 |
| 2 | M | 8503 | 5760 | 292 | 67.74 | 3.43 | 19.99 | 40.99 |
| 3 | M | 9202 | 6274 | 298 | 68.18 | 3.24 | 16.67 | 44.27 |
| 4 | M | 9021 | 6156 | 298 | 68.24 | 3.30 | 20.47 | 36.47 |
| 5 | M | 9693 | 6703 | 298 | 69.15 | 3.07 | 19.63 | 40.99 |
| 6 | M | 8469 | 5611 | 298 | 66.25 | 3.52 | 19.15 | 38.13 |
| 7 | M | 9524 | 6650 | 295 | 69.82 | 3.10 | 21.46 | 39.83 |
| 8 | M | 8946 | 6096 | 297 | 68.14 | 3.32 | 19.12 | 40.29 |
| 9 | M | 9343 | 6378 | 298 | 68.27 | 3.19 | 16.91 | 44.48 |
| 10 | M | 9531 | 6659 | 297 | 69.87 | 3.12 | 21.48 | 38.22 |
| 11 | M | 8731 | 5904 | 297 | 67.62 | 3.40 | 20.19 | 43.18 |
| 12 | M | 8331 | 5569 | 298 | 66.85 | 3.58 | 17.51 | 42.72 |
| 13 | M | 8708 | 5939 | 287 | 68.20 | 3.30 | 18.94 | 41.17 |
| 14 | M | 8790 | 6122 | 288 | 69.65 | 3.28 | 19.17 | 42.14 |
| 15 | M | 8962 | 6193 | 290 | 69.10 | 3.24 | 18.29 | 45.97 |
| 16 | R | 8863 | 5977 | 298 | 67.44 | 3.36 | 19.71 | 39.51 |
| 17 | R | 8459 | 5566 | 298 | 65.80 | 3.52 | 15.12 | 49.52 |
| 18 | R | 9456 | 6513 | 299 | 68.88 | 3.16 | 19.27 | 40.40 |
| 19 | R | 9261 | 6252 | 299 | 67.51 | 3.23 | 17.63 | 42.30 |
| 20 | R | 8547 | 5781 | 297 | 67.64 | 3.47 | 19.00 | 41.43 |
| 21 | R | 9159 | 6268 | 298 | 68.44 | 3.25 | 17.89 | 41.63 |
| 22 | R | 9310 | 6386 | 297 | 68.59 | 3.19 | 17.99 | 43.05 |
| 23 | R | 8671 | 5876 | 297 | 67.77 | 3.43 | 17.15 | 44.52 |
| 24 | R | 9107 | 6201 | 299 | 68.09 | 3.28 | 17.56 | 42.59 |
| 25 | R | 8096 | 5218 | 297 | 64.45 | 3.67 | 16.47 | 40.49 |
| 26 | R | 9086 | 6169 | 297 | 67.90 | 3.27 | 19.29 | 39.07 |
| 27 | R | 7526 | 4789 | 295 | 63.63 | 3.92 | 16.61 | 40.15 |
| 28 | R | 9170 | 6310 | 296 | 68.81 | 3.23 | 18.74 | 40.69 |
| 29 | R | 8369 | 5733 | 291 | 68.50 | 3.48 | 19.85 | 39.89 |
| 30 | R | 8136 | 5486 | 293 | 67.43 | 3.60 | 17.96 | 41.87 |
